# SELPLG Expression Was Potentially Correlated With Metastasis and Prognosis of Osteosarcoma

**DOI:** 10.3389/pore.2022.1610047

**Published:** 2022-01-26

**Authors:** Bingqi Wang, Yufu Sun

**Affiliations:** Department of Orthopedic Surgery, Tianjin First Central Hospital, Tianjin, China

**Keywords:** biomarker, prognosis, metastasis, osteosarcoma, SELPLG

## Abstract

**Background:** Osteosarcoma (OS) is the most prevalent malignant primary bone tumor in children. Selectin P ligand gene (SELPLG) has been studied in several cancers. Our research aimed to explore the role of SELPLG in OS.

**Methods:** All OS patient data was obtained from TARGET and GEO databases. Differential expression analyses were conducted in limma package of R. Functional analyses included GO and KEGG enrichment analyses. Immune cell infiltration analysis was done in CIBERSORT software. The overall survival was calculated using survival and survminer package of R.

**Results:** Significantly lower SELPLG expression was observed in metastatic OS samples compared with non-metastatic OS samples, both in TARGET and in GSE21257. Low SELPLG expression was an independent undesirable prognostic factor for OS patients, in both TARGET and GEO datasets. Totally 62 differentially expressed gene (DEG) overlaps were found between high SELPLG vs. low SELPLG and non-metastatic vs. metastatic OS samples, affecting metastases and thereby influencing the prognosis, which were significantly enriched in 40 GO and six KEGG terms. Five types of immune cells were significantly differentially infiltrated between high and low SELPLG expression OS patients.

**Conclusion:** SELPLG is closely correlated with metastases and prognosis of OS patients. The OS patients with low SELPLG expression have relatively poorer prognosis and SELPLG is a potential prognostic biomarker for OS.

## Introduction

Osteosarcoma (OS), as an aggressive tumor in skeletal system, is the most prevalent malignant primary bone tumor in children, young adults and the elders [1]. Generally, the OS mainly occurs in the metaphysis regions of long bones [2]. Over the past decades, the survival rates of non-metastatic OS patients have significantly increased to 65–75% [3], but the overall 5-years survival rate of patients with metastases and relapsed OS are approximately 30 and 15%, respectively [4, 5], implying an undesirable prognosis. Many OS patients have detectable metastases at presentation, and the most common sites of OS metastases include lung (85%) and bone [6, 7]. Moreover, the great heterogeneity in OS patients limits the improvement of diagnosis and prognosis, which makes it difficult to identify reliable biomarkers and the cell type causing OS [8]. Although the pathogenesis of OS is quite complicated, especially from a molecular aspect, some previous studies give us important inspiration [7]. Aberrant expressions of several genes show vital effects on OS, such as TP53 [9], Rb [10], c-myc [11], etc. Accordingly, further investigation in OS is urgently needed to better understand the molecular mechanisms of OS tumorigenesis and provide more alternatives for clinical therapeutic strategies, in order to improve the prognosis of OS patients.

Selectin P ligand gene (SELPLG), also known as CD162 and PSGL-1, encodes P-selectin glycoprotein ligand 1 (PSGL-1) [12], which is involved in immune cell trafficking and the regulation of myeloid cell immune responses [13]. As a member of the selectin family, SELPLG mostly expresses in some immune or inflammatory cells [14]. SELPLG deficiency has been documented to influence the myeloid cell differentiation and lymphocyte maturation, thus it is important in cell differentiation [15]. Additionally, SELPLG has been investigated in many human diseases. For instance, SELPLG is identified as a novel acute respiratory distress syndrome (ARDS) susceptibility gene and a promising therapeutic target in ARDS [12]. Recently, SELPLG has been demonstrated to act as an immune checkpoint regulator, which might be novel therapeutic target in cancer [13]. It has been reported to mediate the development and chemotherapy resistance of acute myeloid leukemia (AML), and blocking the binding of SELPLG to E-selectin is probably a new target [16]. Moreover, SELPLG has been studied in several kinds of cancers as potential diagnostic or prognostic biomarkers, such as colorectal cancer (CRC) [17], head and neck squamous cell carcinoma (HNSCC) [18], anaplastic large T-cell lymphoma (ALCL) [19], uveal melanoma [20], and so on. In The Human Protein Atlas database (https://www.proteinatlas.org/), SELPLG is a favorable factor in most recorded tumors (such as cervical cancer, thyroid cancer, and so on). Whereas, to the best of our knowledge, few reports have focused on SELPLG in OS. Consequently, we hope that our research would provide more insights in the potential role of SELPLG in OS patients.

Herein, the purpose of our study was to explore the potential role of SELPLG in the metastasis and prognosis of OS patients utilizing the effective bioinformatic tools, based on the publicly available data in TARGET and GEO databases. The final results might be meaningful to improve the prognosis of OS patients in future clinical cases.

## Materials and Methods

### Data Collection

The mRNA expression profile and clinical information of 88 OS patients was obtained from the Therapeutically Applicable Research To Generate Effective Treatments (TARGET) (https://ocg.cancer.gov/programs/target) database, of which 84 OS patients with complete survival information were further analyzed. The detailed patient information was listed in Table 1.

**TABLE 1:** Clinicopathological characteristics of OS patients from TARGET database.

Characteristics		Patients(N = 84)	
		NO.	%
Sex	Female	37	44.05
	Male	47	55.95
Age	≤14 (Median)	44	52.38
	>14 (Median)	40	47.62
Race	White	51	60.71
	Asian	6	7.14
	Black or African American	7	8.33
	Unknown	20	23.81
Disease at diagnosis	Metastatic	21	25.00
	Non-metastatic	63	75.00
Primary tumor site	Arm/hand	6	7.14
	Leg/foot	76	90.48
	Pelvis	2	2.38
Vital status	Dead	27	32.14
	Alive	57	67.86

Additionally, the mRNA and clinical data in other two datasets were also downloaded from the Gene Expression Omnibus (GEO) (https://www.ncbi.nlm.nih.gov/geo/) database. Dataset GSE21257 [20, 21] included 53 OS samples, mRNA data of which was detected in Illumina human-6 v2.0 expression beadchip. GSE16091 [22] contained 34 OS samples, mRNA profile was detected using Affymetrix Human Genome U133A Array. The clinical information was summarized in Supplementary Table S1.

### Differentially Expressed Genes

We have done the differentially expressed genes (DEGs) analyses in limma package of R language (version 4.0.2, the same below). The DEG screening criteria was |Log_2_FC| >1 and *p* value ≤ 0.05.

### Functional Enrichment Analyses

The screened DEGs were then subjected to the Gene ontology (including Biological Process (BP), Molecular Function (MF), Cellular Component (CC)) and Kyoto Encyclopedia of Genes and Genomes (KEGG) enrichment analyses in clusterProfiler package of R [23]. The terms with *p* < 0.05 were considered significantly enriched.

### Immune Cell Infiltration

The relative proportion of 22 kinds of immune cells in each OS sample was calculated in CIBERSORT [24] software. The composition of immune infiltrating cells was characterized by the 547 preset barcode genes according to the deconvolution algorithm, on a basis of the gene expression matrix. The sum of all estimated immune cells’ proportion in each sample is equal to 1.

### Statistical Analyses

The overall survival of various groups was calculated using survival and survminer package of R (https://CRAN.R-project.org/package=survminer) based on the Kaplan-Meier method. The significance of difference was tested using log-rank. The immune cell infiltration difference among different groups was determined by Wilcoxon signed-rank test (*p* < 0.05 was significance threshold). All statistical analyses utilized R software v3.5.2.

## Results

### Low SELPLG Expression Was Correlated With the OS Metastases

**FIGURE 1:**
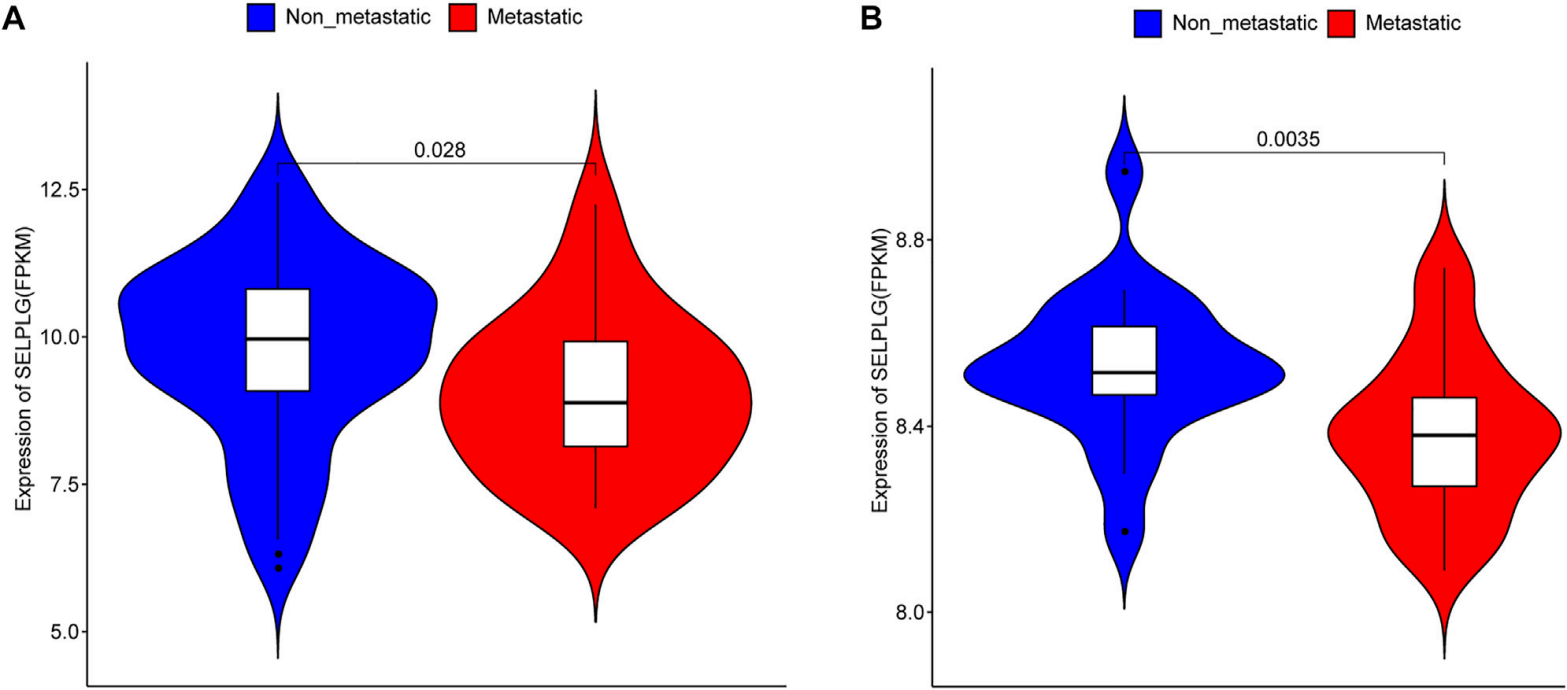
SELPLG expression in metastatic and non-metastatic OS samples. **(A)** SELPLG expression in TARGET database. **(B)** SELPLG expression in GSE21257 dataset.

To explore the association between SELPLG and OS metastases, we firstly compared the expression of SELPLG in metastatic OS and non-metastatic OS samples (patients with or without metastasis at diagnosis) in TARGET database (Table 1). The results showed that the expression of SELPLG in metastatic OS samples was significantly lower than that in non-metastatic OS samples (Figure 1A). Moreover, in GSE21257 dataset (patients with or without metastasis in 5 years after diagnosis), significantly lower SELPLG expression was also observed in metastatic OS samples compared with the non-metastatic OS samples (Figure 1B). Our results suggested that low SELPLG expression might be related to the metastases of OS.

### The OS Patients With Low SELPLG Expression had an Undesirable Prognosis

**FIGURE 2:**
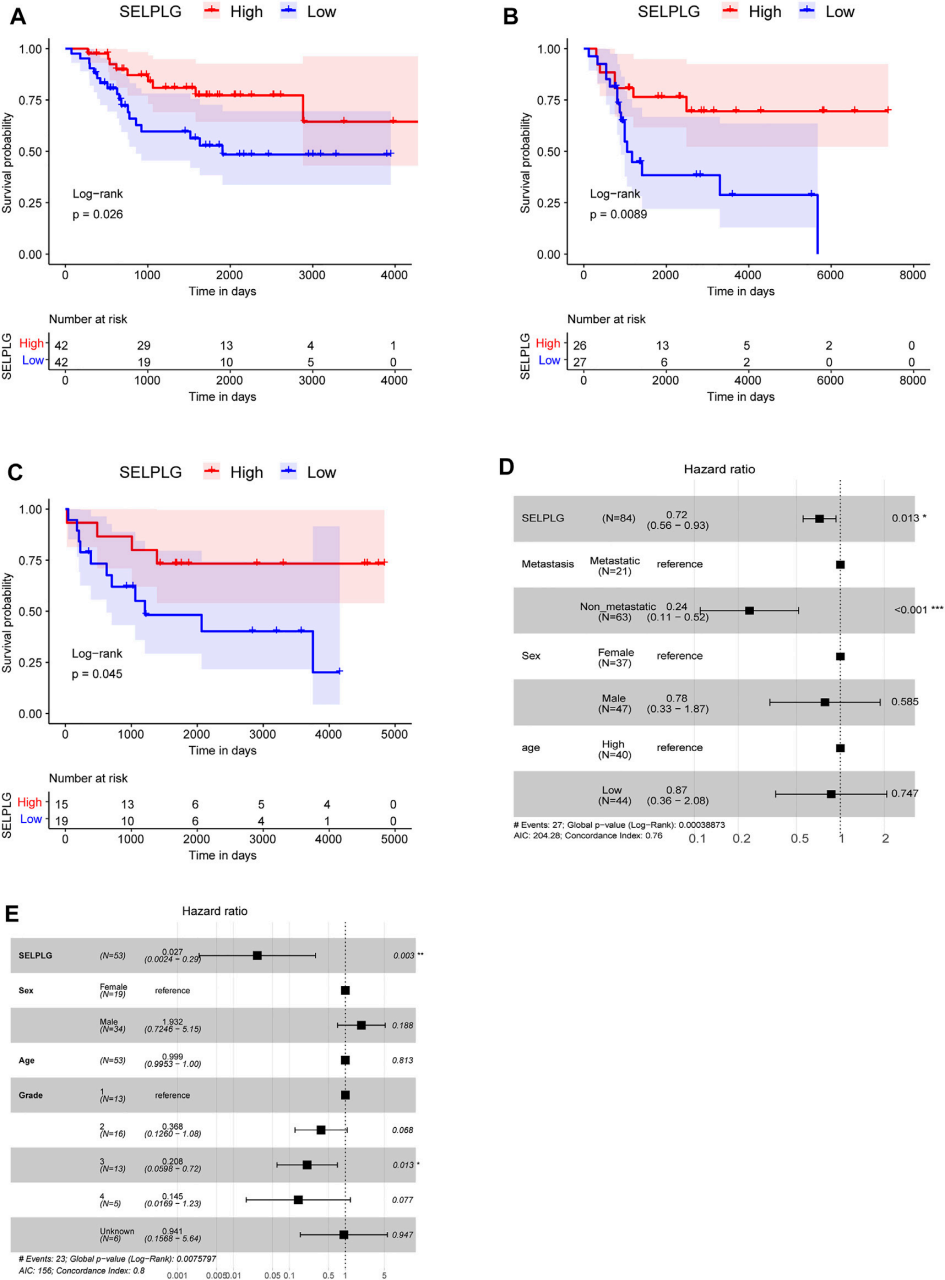
The OS patients with low SELPLG expression had a worse prognosis. **(A–C)** Kaplan-Meier survival curves of high and low SELPLG expression OS samples in TARGET, GSE21257, and GSE16091, respectively. *p* value was determined by log-rank test. **(D,E)** Multivariate Cox regression analysis results in TARGET database and GSE21257 dataset, separately. Compared with reference samples, Hazard ratio (HR) > 1 means a higher risk of death, HR < 1 means a lower risk of death.

Subsequently, all OS samples in TARGET database were divided into high and low SELPLG expression groups according to the median, in order to study the influence of SELPLG expression on the prognosis of OS patients. After survival analyses, we found that high SELPLG expression OS patients had better overall survival compared with low SELPLG expression OS patients (*p* = 0.026, Figure 2A). Then the survival analyses were also conducted in two GEO datasets GSE21257 and GSE16091, the results showed a similar tendency (Figures 2B,C). Our findings indicated that the OS patients with low SELPLG expression probably had a poor prognosis.

A multivariate Cox regression analysis, including age (age > 14 vs. age ≤ 14), sex (female vs. male), metastasis (metastasis vs. no metastasis), grade, and SELPLG (high SELPLG expression vs. low SELPLG expression), was performed to determine whether SELPLG expression is an independent prognostic indicator for OS. Both in TARGET database (HR = 0.72, 95% CI: 0.56–0.94, *p* = 0.014, Figure 2D) and GSE21257 dataset (HR = 0.027, 95% CI: 0.0024–0.29, *p* = 0.003, Figure 2E), SELPLG expression was significantly correlated with overall survival of OS patients. The OS patients with high SELPLG expression had relatively lower death risk, and high SELPLG expression was a protective prognostic factor for OS. On the contrary, low SELPLG expression was a poor prognostic indicator for OS patients.

### SELPLG Expression Might Affect the Prognosis by Mediating the Metastasis in OS Patients

**FIGURE 3:**
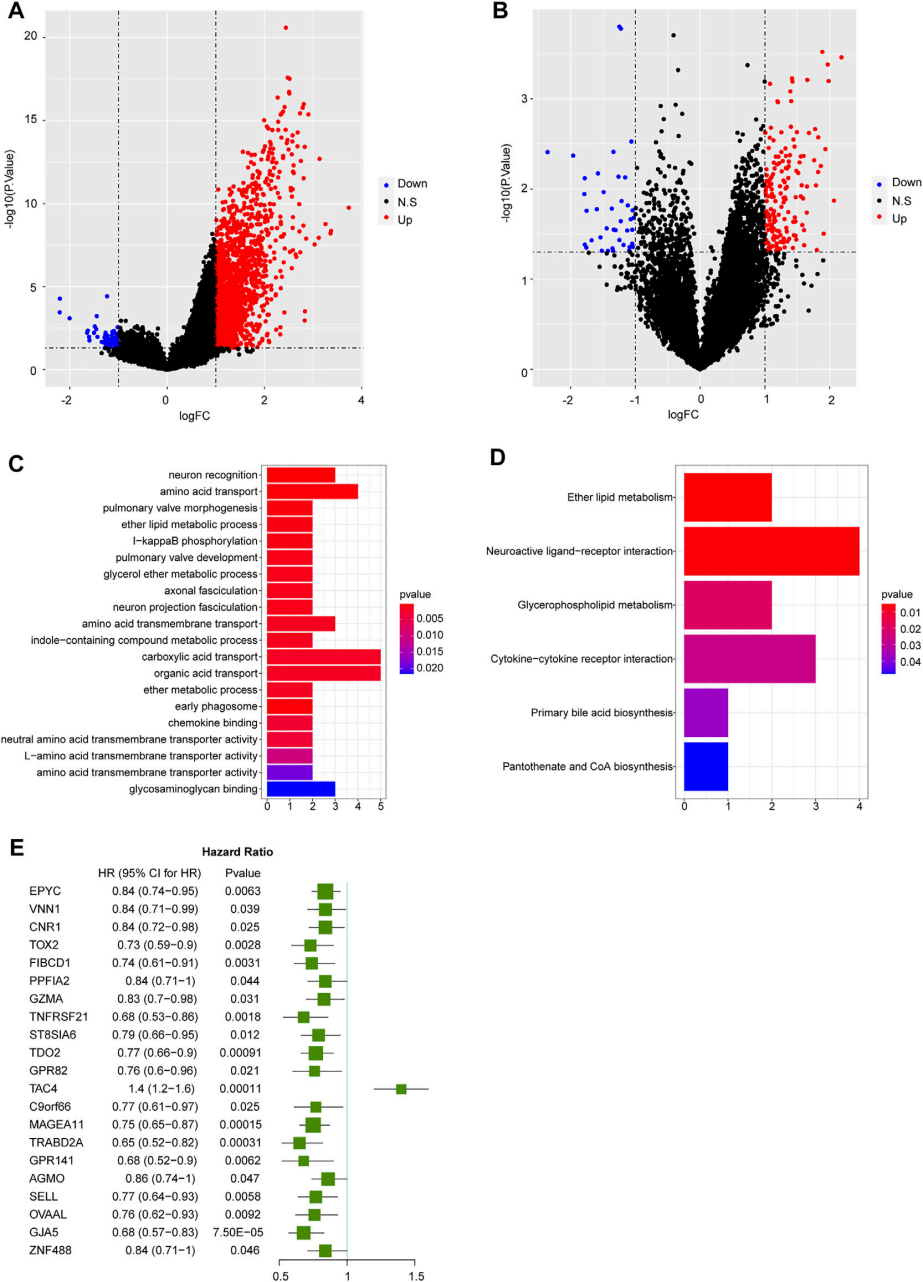
The results of differential expression analyses and functional enrichment. **(A)** DEGs identified between high and low SELPLG expression OS samples. **(B)** DEGs identified between metastatic and non-metastatic OS samples. **(C)** The top 20 most significantly enriched GO terms. **(D)** Six significantly enriched KEGG pathways. **(E)** 21 genes were significantly related to the prognosis of OS patients.

In TARGET database, totally 1730 DEGs were identified in high SELPLG expression OS patients compared with low SELPLG expression OS patients, including 1,665 up-regulated genes and 65 down-regulated genes (Figure 3A). Additionally, we have identified 209 DEGs in non-metastatic OS samples compared with metastatic OS samples, of which 169 genes were up-regulated and 40 genes were down-regulated (Figure 3B). To further find genes influenced by differential SELPLG expression, 1730 DEGs and 209 DEGs were cross-analyzed and 62 DEG overlaps were found, which might influence the metastases of OS, leading to differential prognoses (Supplementary Table S2). We suspected that these 62 DEGs were affected by differential SELPLG expression, and influenced the metastases of OS.

We then conducted a functional enrichment on the 62 DEGs. The 62 DEGs were significantly enriched in 40 GO terms and six KEGG pathways. The most significantly enriched 20 GO terms and all six KEGG pathways were displayed in Figures 3C,D. All enrichment results were displayed in Supplementary Table S3. Moreover, basing on these 62 DEGs, an univariate Cox regression analysis was conducted to explore their potential association with the prognosis of OS patients. Our results suggested that 21 genes were significantly related to the prognosis of OS patients (Figure 3E). Among which, except for TAC4, the rest 20 prognosis related genes were all significantly highly expressed in non-metastatic and high SELPLG expression OS samples. Therefore, we suspected that high SELPLG expression might reduce the risk of metastasis in OS patients, thereby leading to a better prognosis.

### Immune Cell Infiltration in OS Patients With Differentially Expressed SELPLG

**FIGURE 4:**
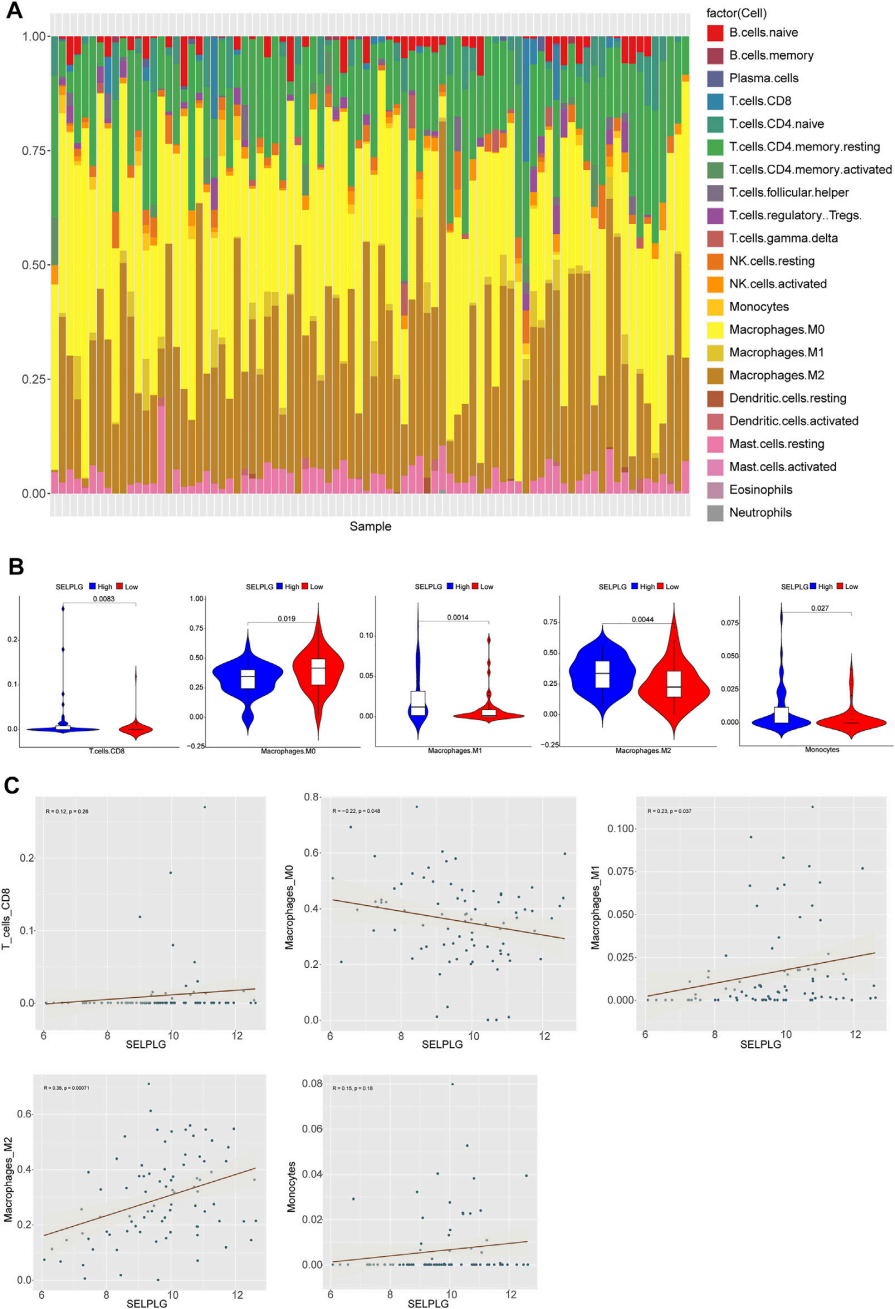
Immune cell infiltration in OS patients with differentially expressed SELPLG. **(A)** The relative infiltration ratios of 22 kinds of immune cells in all 84 OS patients. **(B)** The infiltration ratios of five kinds of immune cells were significantly different between high and low SELPLG expression OS patients. **(C)** The correlation between CD8 T cells, M0, M1, M2 Macrophages, Monocytes’ infiltration and SELPLG expression in OS samples, respectively.

Based on CIBERSORT method and LM22 feature matrix, the immune infiltration differences of 22 kinds of immune cells were estimated between high and low SELPLG expression OS samples. The immune cell infiltration in 84 OS patients was shown in Figure 4A, and relative infiltration ratios of immune cells in various OS patients were differential, indicating the differentially inherent characteristics. Additionally, we found that five kinds of immune cells’ infiltration (including Monocytes, M0, M1, M2 Macrophages, and CD8 T cells) were significantly different between high and low SELPLG expression OS patients (Figure 4B), which might be potential factors affecting the differential prognoses of high and low SELPLG expression OS patients. Moreover, the correlation analysis was done to explore the potential association between SELPLG expression and these immune cells’ infiltration. The correlation between CD8 T cells, M0, M1, M2 Macrophages, Monocytes and SELPLG expression in OS samples were shown in Figure 4C, respectively. There was a significantly positive correlation between M1, M2 Macrophages and SELPLG expression, while a negative correlation between M0 Macrophages and SELPLG expression in OS samples (Figure 4C).

## Discussion

Recently, the crucial role of SELPLG in tumor metastasis has been demonstrated [25]. In this work, we have explored the association between SELPLG and the metastasis, prognosis of OS patients using the powerful bioinformatic tools, basing on the data in TARGET and GEO databases. We found that low SELPLG expression was closely associated with the metastases of OS patients, and OS patients with low SELPLG expression had relatively worse prognosis.

Compared with non-metastatic OS patients, metastatic OS patients usually have relatively poorer outcome in most clinical cases [4, 5]. Firstly, we found that significantly lower SELPLG expression was observed in metastatic OS samples compared with non-metastatic OS samples, both in TARGET and in GSE21257 dataset, indicating low SELPLG expression might be related to the metastases of OS. In colorectal cancer (CRC), it has been evidenced that SELPLG deficiency could render intestinal tissue more vulnerable to grow colorectal tumors [14]. Moreover, SELPLG was indicated to be downregulated in primary human AML M2 t(8;21)+ leukemia cells, besides RUNX1/ETO-mediated SELPLG suppression would probably reduce the cell adhesion of t(8;21)+ acute myeloid leukemia cells [26]. Cell adhesion might have impact on the move of leukemia cells [27], while more direct details among SELPLG, cell adhesion, and metastasis remained to be unclear. Whereas, SELPLG showed strong expression in primary effusion lymphoma (PEL), and it was vital for cell migration and chemotaxis [28], the expression of which was converse in OS in our research. The above evidence suggests that the role of SELPLG in different tumors might be different, more details of which are still unclear. Moreover, based on the results of survival analyses and multivariate Cox regression analyses, low SELPLG expression was an independent undesirable prognostic factor for OS patients, in both TARGET database and GEO datasets. It has been documented that the copy number of SELPLG was reported as a biomarker to differentiate subtypes of AML [29]. SELPLG was also identified in HNSCC and ALCL as predictor or potential therapeutic target [18, 19]. We have firstly reported SELPLG as a prognostic biomarker for OS, which might serve as a reference factor for OS prognosis prediction in the future.

Additionally, there were 1730 DEGs between high and low SELPLG expression OS patients, and 209 DEGs between non-metastatic and metastatic OS samples. To further identify the DEGs affecting metastases and then influencing the prognosis, 62 DEG overlaps were found. The subsequent functional analyses showed they were significantly enriched in 40 GO terms and six KEGG pathways. Neuroactive ligand-receptor interaction pathway has been suggested to be implicated in tumorigenesis of OS, which might be affected by abnormal DNA methylation [30, 31]. The mesenchymal stem cells (MSCs) might provide an advantageous source of microenvironments for OS cells partly through Cytokine-cytokine receptor interaction pathway [32]. However, some metabolism and biosynthesis related pathways, such as Ether lipid metabolism pathway, Glycerophospholipid metabolism pathway, Primary bile acid biosynthesis pathway, and Pantothenate and CoA biosynthesis pathway, have not been concretely studied in OS, which should be further studied.

Furthermore, given to the important role of selectins in tumor metastatic spread [33, 34], the immune cell infiltration was also analyzed between high and low SELPLG expression OS patients. Five kinds of immune cells, including Monocytes, M0, M1, M2 Macrophages, and CD8 T cells, were significantly differentially infiltrated between high and low SELPLG expression OS patients. The endogenous PSGL-1 encoded by SELPLG would facilitate the recruitment of monocytes to metastasize tumor cells, then contributing to metastasis would result in decreased survival [35], which supported our findings indirectly. However, the causal connection between the monocyte recruitment and SELPLG expression remains unclear in OS samples, which needs to be clarified via deepening exploration. Various macrophages M0, M1 (inflammatory macrophages), and M2 (alternative macrophages) have been evidenced to have fundamental role in the pathogenesis of OS [36]. Tumor-infiltrating macrophages are documented to orchestrate many aspects of OS and increased infiltration of M2 is correlated with the metastasis of OS and poor prognosis [37]. The above evidences imply that these immune cells’ infiltration does directly or indirectly affect the metastasis and undesirable prognosis of OS, which deserves further exploration in our future researches.

To summarize, we have firstly investigated the possible role of SELPLG in OS patients. Our findings indicate that SELPLG is closely correlated with the metastases and prognosis of OS patients. The OS patients with low SELPLG expression have relatively poorer prognosis and SELPLG is a potential prognostic biomarker for OS.

## Data Availability

The original contributions presented in the study are included in the article/Supplementary Material, further inquiries can be directed to the corresponding author.

## Abbreviations

OS, osteosarcoma; SELPLG, selectin P ligand gene; ARDS, acute respiratory distress syndrome; AML, acute myeloid leukemia; CRC, colorectal cancer; HNSCC, head and neck squamous cell carcinoma; ALCL, anaplastic large T-cell lymphoma; TARGET, therapeutically applicable research to generate effective treatments; GEO, gene expression omnibus; DEGs, differentially expressed genes; BP, biological process; MF, molecular function; CC, cellular component; MSCs, mesenchymal stem cells.
